# Geo-Demographic and Socioeconomic Determinants of Diagnosed Hypertension among Urban Dwellers in Ibadan, Nigeria: A Community-based Study

**DOI:** 10.21203/rs.3.rs-3692586/v1

**Published:** 2023-12-20

**Authors:** Mayowa Owolabi, Olalekan Taiwo, Joshua Akinyemi, Ayodeji Adebayo, Oluwafemi Popoola, Rufus Akinyemi, Onoja Akpa, Paul Olowoyo, Akinkunmi Okekunle, Ezinne Uvere, Chukwuemeka Nwimo, Omotolani Ajala, Olayinka Adebajo, Adewale Ayodele, Salami Ayodeji, Oyedunni Arulogun, Olanrewaju Olaniyan, Richard Walker, Carolyn Jenkins, Bruce Ovbiagele

**Affiliations:** Center for Genomic and Precision Medicine, University of Ibadan, Nigeria; University of Ibadan, Nigeria; University of Ibadan; University of Ibadan; University of Ibadan; University of Ibadan, College of Medicine; University of Ibadan; University of Ibadan; University of Ibadan; University of Ibadan; University of Ibadan; University of Ibadan; University of Ibadan; Northumbria Healthcare NHS Foundation Trust; Medical University of South Carolina; University of California San Francisco

**Keywords:** High blood pressure, Noise, Faith-based organisations, Spatial analysis, Ibadan

## Abstract

**Background:**

The relationship between diagnosed high blood pressure (HBP) and proximity to health facilities and noise sources is poorly understood. We investigated the relationship between proximity to noise sources, sociodemographic and economic factors, and diagnosed HBP in Ibadan, Nigeria.

**Methods:**

We investigated 13,531 adults from the African Rigorous Innovative Stroke Epidemiological Surveillance (ARISES) study in Ibadan. Using a Geographic Information System (GIS), the locations of healthcare facilities, pharmaceutical shops, bus stops, churches, and mosques were buffered at 100m intervals, and coordinates of persons diagnosed with HBP were overlaid on the buffered features. The number of persons with diagnosed HBP living at every 100m interval was estimated. Gender, occupation, marital status, educational status, type of housing, age, and income were used as predictor variables. Analysis was conducted using Spearman rank correlation and binary logistic regression at p<0.05.

**Results:**

There was a significant inverse relationship between the number of persons diagnosed with HBP and distance from pharmaceutical shops (r=−0.818), churches (r=−0.818), mosques (r=−0.893) and major roads (r=−0.667). The odds of diagnosed HBP were higher among the unemployed (AOR=1.58, 95% CI: 1.11–2.24), currently married (AOR=1.45, CI: 1.11–1.89), and previously married (1.75, CI: 1.29–2.38). The odds of diagnosed HBP increased with educational level and age group.

**Conclusion:**

Proximity to noise sources, being unemployed and educational level were associated with diagnosed HBP. Reduction in noise generation, transmission, and exposure could reduce the burden of hypertension in urban settings.

## 1. Introduction

High blood pressure (HBP) is defined as systolic blood pressure ≥ 140 mmHg and/or diastolic blood pressure ≥ 90 mm Hg. ^1^ HBP affects more than 1 billion people worldwide, the majority of whom live in low- and middle-income countries.^2^ Hypertension, high blood pressure (HBP) is the most important risk factor for death and cardiovascular diseases. ^3^ Long exposure to noise > 90 dBA has been identified as one of the drivers of HBP. ^4,5^

In Europe, traffic noise is responsible for 1.7 million cases of hypertension.^6^ Long-term or short-term effects of exposure to environmental noise may affect the autonomic and endocrine systems resulting in changes in BP regulation that then contribute to an increased risk of cardiovascular diseases.^5^ A meta-analysis of 24 studies revealed that road traffic noise is associated with an elevated risk of HBP.^7^ Traffic-related noise at night causes fragmentation of sleep, the elevation of stress hormone levels, and oxidative stress which ultimately promote the development of vascular (endothelial) dysfunction, and HBP.^5^

Previous studies on the impacts of noise on CVD have focused on industrial exposure with limited attention to noise from religious organizations. ^8^ In a study of 200 mosques in Riyadh there was significantly greater noise outside, compared to inside the prayer rooms. Similarly, noise levels from places of worship were higher than those set by Jordanian limits during day and night time. ^9^ The relationship between HBP and distance from roads and religious centres (churches and mosques), which are noise sources, has not been explored, in Nigeria, where noise from religious centres and other public facilities is unregulated.

Therefore, we investigated the relationship between proximity to noise sources and the likelihood of being diagnosed with HBP in a largely indigenous population in Ibadan, Nigeria. In addition, we also examined the relationship between the number of persons diagnosed with HBP and the distance to potential BP monitoring facilities (healthcare facilities and pharmaceutical shops).

## 2. Material and Methods

### 2.1 Data Collection

Door-to-door survey was used to collect data between 11 October 2021 and 29 August 2022 during the baseline phase of the African Rigorous Innovative Stroke Epidemiological Surveillance (ARISES) project.^10^ A very high-resolution satellite image (Google Earth) showing all the buildings in the demographic surveillance sites (DSS) was converted to PDF format and loaded to Avenza Maps software (https://www.avenza.com/avenza-maps/) for ease of ground navigation to respective buildings. This ensured near-total case inclusion and accurate denominator population. Enumerators interviewed household head or his/her proxy and collected data on demographic characteristics, stroke knowledge, stroke status, ownership of household assets and information on diagnosis of stroke high blood pressure and other related co-morbidity. Data was stored electronically using REDCap mobile app.^11^ Diagnosed HBP was defined as previous diagnosis of high blood pressure (BP ≥ 140/90 by a trained healthcare worker using standard protocol and/or current treatment for HBP). Furthermore, data on the locations of healthcare facilities, pharmaceutical shops, churches, mosques, and bus stops were digitized from Google Maps and validated during the data collection survey.

### 2.2 Data Analysis

The urban DSS data were filtered to select only those that had previously been diagnosed with HBP. The geographic coordinates of all these persons were plotted in ArcGIS software. Similarly, the locations of pharmaceutical shops, healthcare facilities, churches, mosques, and bus stops were also plotted and overlaid on the locations of the persons diagnosed with HBP to assess the relationship between distance from these healthcare facilities and noise-generating centres.

The Average Nearest Neighbour (Rn) analysis was used to identify the distributional pattern of persons diagnosed with HBP, healthcare facilities (pharmaceutical/patient medicine shops, and hospitals/clinics), and noise-generating centres (churches, mosques, and bus stops).^12–14^ The Rn is calculated as the observed average distance divided by the expected average distance.^12^ If the average distance is less than the average for a hypothetical random distribution, the distribution of the features is considered clustered, however, if it is greater than a hypothetical random distribution, it is considered dispersed, while if it is 1, it is regarded as random.^14^ The hotspot of persons diagnosed with HBP was analysed using Kernel Density Estimation (KDE) ^15^. The method identified localities with higher concentrations of persons diagnosed with HBP.^12^

A total of 1,510 persons previously diagnosed with HBP based on their response in the questionnaire were selected from 13,531 respondents. The coordinates of diagnosed HBP patients were overlaid on the road network, the location of churches, mosques, health facilities, and pharmaceutical shops. The number of persons diagnosed with HBP at every 100 meters from each healthcare facility types and noise-generating centres types were enumerated using series of non-overlapping 100 meters multiple ring buffers.^16^ ArcGIS software was used for all the spatial analyses, while Spearman rank Correlation was used to assess the statistical relationship between distance from healthcare facilities, noise-generating facilities and the number of persons diagnosed with HBP. Binary logistic regression was used to assess the association between the demographic, socioeconomic, and housing variables and the likelihood of HBP diagnosis among the entire sample of 13531 respondents at ρ < 0.05. The dependent variable is a binary categorical variable indicating whether a person reported to have been diagnosed with HBP or otherwise. The regression results were presented as odds ratios (OR) together with their 95% confidence intervals (CIs). All comparisons were considered to be statistically significant at p < 0.05 level.

## 3. Results

### 3.1 Distributional Pattern of Diagnosed HBP Patients, Health Care Facility and Noise Generating Locations.

**Figure 1:**
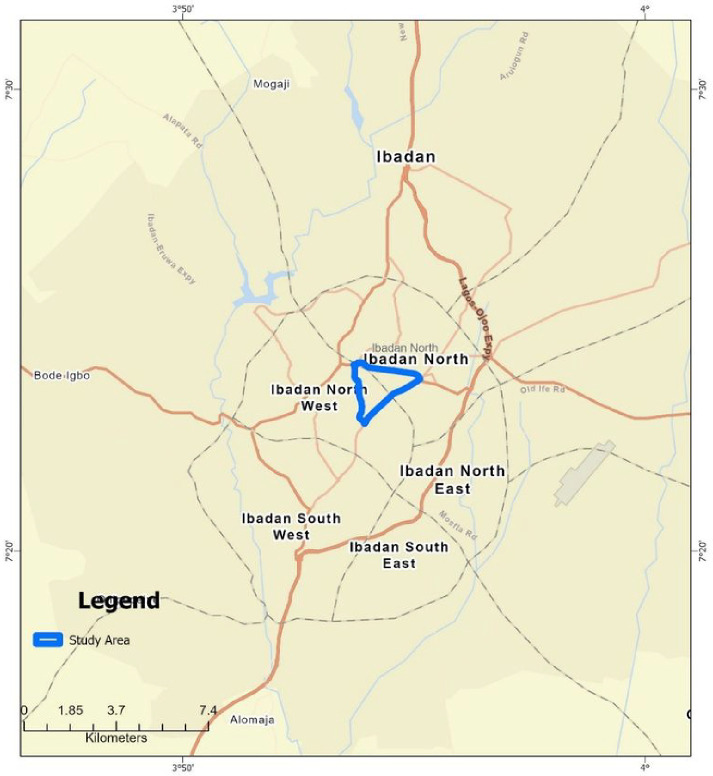
The Study Administrative Wards within the Ibadan Metropolitan Area, Oyo State.

In all, 1510 (11.16%) of the 13531 respondents in the urban DSS (Fig. 1) had been diagnosed with HBP. The spatial distribution analysis reveals distinct patterns in the locations of individuals diagnosed with HBP and pharmaceutical shops. The locations of individuals diagnosed with HBP and the location pharmaceutical shops are clustered in the urban DSS (Table 1).^14^ Conversely, the distribution of hospitals/Clinics, churches, and bus stops are dispersed. However, mosques are randomly distributed (Table 1).

**Figure 2:**
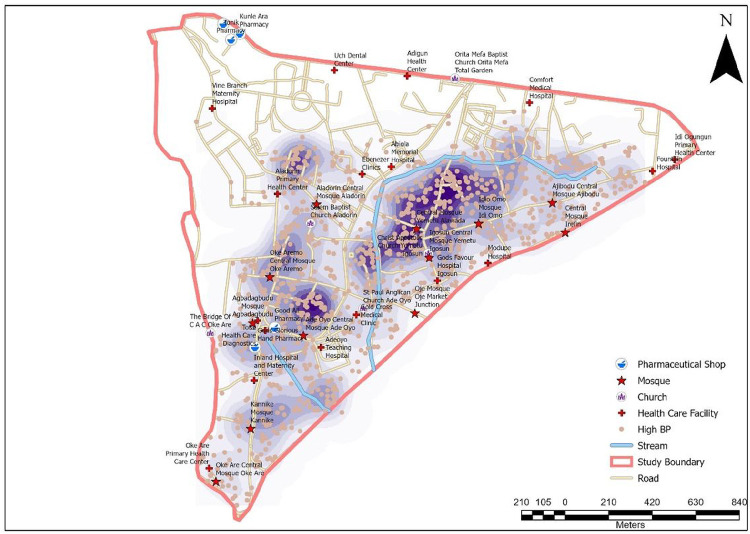
Kernel Density Estimation of Self-Reported HBP

The highest concentration of persons diagnosed with HBP are in localities such as Jayode Hospital area, Ile-Aloko Mosque area, The Bride of Christ Apostolic Church, Ayegbami Mosque, Hardex Logistic area, Olounloleru Mosque, The Hope of Heaven Christ Family Church, and Highland Specialist Hospital and Somide Creative Agency, etc. (Fig. 2).

### 3.2 Proximity to Healthcare Facilities and Pharmaceutical Shops and Diagnosed HBP

The average distance to healthcare facilities among persons with diagnosed HBP was significantly higher (214.35 ± 91.13m) than for those without diagnosed HBP (205.44 ± 94.27m F_(1,13464)_ = 12.037, p = 0.001). However, there was no significant relationship between the number of persons diagnosed with HBP and distance from healthcare facilities (r = 0.300, p = 0.312). Thus, while the average distance travelled to hospitals varied between those diagnosed and those not diagnosed with HBP, nonetheless, there was no significant relationship between the number of persons diagnosed with HBP and distance from healthcare facilities. The average distance to pharmaceutical shops for persons diagnosed with HBP was slightly but not significantly lower (737.11 ± 455.30m) than for those without diagnosed HBP (740.85 ± 479.06m; F_(1,13464)_ = 0.082, p = 0.774). However, there was a significant negative relationship between distance from pharmaceutical shops and the number of persons with diagnosed HBP (r = 0-.818, p = 0.001).

### 3.3 Proximity to Churches and Mosques and Diagnosed HBP

The average distance to churches was significantly lower among persons with diagnosed HBP (344.39 ± 194.44 meters) compared to those without diagnosed HBP (356.93 ± 212.36 meters; F_(1,13464)_ = 4.754, p = 0.029). There was a significant inverse relationship between distance from churches and the number of persons with diagnosed HBP (r=−0.818, p = 0.001). There was no significant difference between the distance from mosques among those with diagnosed HBP (178.29 ± 92.44m) and those without (179.11 ± 93.93; F_(1,13464)_ = 0.106, p = 0.745). However, there was a significant inverse relationship between distance from mosques and the number of persons with diagnosed HBP (r=−0.893, p = 0.003).

### 3.4 Proximity to Road Infrastructure and Diagnosed HBP

The average distance from bus terminals was significantly lower 420.27 ± 175.40meters in persons with diagnosed HBP than persons without (438.52 ± 173.52; F_(1,13464)_ = 14.775, p < 0.0001). However, there was no significant relationship between the number of persons with diagnosed HBP and distance from bus terminals (r=−0.389, p = 0.133). There was no significant difference between distance from non-major roads among those with diagnosed HBP (33.00 ± 25.42m) and those without (33.82 ± 24.83m; F_(1,13464)_ = 1.481, ρ = .224). The average distance from major roads among persons with diagnosed HBP (353.16 ± 189.00m) was significantly higher than for those without diagnosed HBP (326.88 ± 196.59; F_(1,13464)_ = 24.132, p < 0.0001). However, there was a significant inverse relationship between distance from major roads and the number of persons with diagnosed HBP (r=−.667, p = .006).

### 3.5 Association between Demographic, Socioeconomic, and Clinical Characteristics with Diagnosed HBP

Persons with diagnosed HBP were significantly older (age: 50.36 ± 17.57 years) than those without diagnosed HBP (39.23 ± 16.10 years; F_(1,13464)_ = 626.147, p < 0.0001). There was no significant difference in mean monthly income between persons diagnosed with HBP (37981.49 ± 95593.89 naira) and those not diagnosed with HBP (36233.87 ± 86496.23 naira; F_(1,13464)_ = 0.533, p = 0.465).

Furthermore, there was no significant difference in proportions of males (2.7%) with diagnosed HBP, compared to females (8.5%) with diagnosed HBP (χ^2^ = 2.085, p = 0.079). Greater percentage (Table 2) of married persons were diagnosed with HBP. Marital status was significantly associated with diagnosed HBP (χ^2^ = 308.067, df = 3, *ρ* < 0.05). Education was also found to be significantly associated with diagnosed HBP (χ^2^ = 164.773, *p* < 0.05), with persons who had no formal education exhibiting the highest percentage and those with primary education exhibiting the lowest percentage (Table 2). There is a significant occupational difference between those with diagnosed HBP (χ^2^ = 107.841, *ρ* < 0.05) and those without, with professionals having the lowest percentage of persons diagnosed with HBP (Table 4).

Regarding health-related variables, diabetes mellitus was significantly associated with diagnosed HBP (χ^2^ = 181.342, p < 0.05), with individuals without diabetes mellitus being less likely to have diagnosed HBP. Those with diagnosed HBP had a significantly higher proportion of high cholesterol than those not diagnosed with HBP (χ^2^ = 38.898, p < 0.0001) or obesity (χ^2^ = 20.801, p < 0.0001). There was a strong association between stroke and number of diagnosed HBP (χ^2^ = 281.253, p < 0.0001), with a greater proportion of respondents who had a stroke also previously diagnosed with HBP (Table 2). Furthermore, tobacco use was significantly associated with diagnosed HBP ((χ^2^ = 19.692, *ρ* < 0.05). Kolanut consumption was significantly associated with diagnosed HBP (χ^2^ = 255.042, *ρ* < 0.05I). Alcohol consumption was significantly associated with diagnosed HBP (χ^2^ = 131.259, p = 0.0001; Table 2).

### 3.3 Socioeconomic Determinants of Diagnosed HBP

The determinants of diagnosed HBP include occupation, marital status, education, housing type, and age. Following adjustment for demographic and socio-economic characteristics, the odds of diagnosed HBP were 58% higher among unemployed persons compared to professionals (AOR = 1.58, CI: 1.11, 2.24). Similarly, the currently married (AOR = 1.45, CI: 1.11, 1.89) and previously married persons (AOR = 1.75, CI: 1.29, 2.38) were more likely to be diagnosed with HBP. The likelihood of HBP diagnosis was highest among those with tertiary education (AOR = 1.64, CI: 1.25, 2.16) relative to those with no formal education. Furthermore, the odds of diagnosed HBP increased with the age of participants (Table 3). The Binary Logistic Regression model correctly classified 88.8% of the cases.

## 4. Discussion

This study sought to examined the association between the location of persons with diagnosed HBP and location of healthcare facilities (pharmaceutical shops, hospitals/clinic) and noise generating facilities (church, mosques and bus stops). Also, we investigated the socioeconomic predictors of diagnosed HBP among the residents. We established that persons with diagnosed HBP were clustered in space. Consequently, some local factors may be responsible for the observed distributional pattern. An overlay of the locations of churches and mosques on the Kernel Density Estimation Map shows that hotspots of persons with diagnosed HBP are within the localities where all the churches and mosques are located. Similarly, the hotspots of persons with diagnosed HBP also aligned with the major roads. Hence, there is a close association between the distribution of persons with diagnosed HBP and the distribution of churches, mosques and major road alignments. However, a relatively disproportional lower number of the pharmaceutical shops are located within the hotspots of persons diagnosed with HBP.

Access to healthcare is constrained by numerous geographically varying factors, including long travel times to healthcare facilities and poor transportation infrastructure.^17^ Most of the hospitals/clinics in the area are primary healthcare facilities that respond to the basic healthcare needs of the people. This perhaps accounts for why there was no significant relationship between distance from hospitals/clinics and the number of persons with diagnosed HBP. However, the distribution of pharmaceutical shops was clustered because they are often located in response to population and this could explain the negative association between distance from pharmaceutical shops and the number of persons with diagnosed HBP. In addition, proximity to pharmaceutical shops could also serve as an impetus to check one’s BP. This is because most of the pharmaceutical shops provide a BP checking service for a small fee to local people.

Persons with diagnosed HBP lived much closer to churches than those without diagnosed HBP. Churches are dispersed and the percentage of persons with diagnosed HBP declined with increasing distance from churches, hence, proximity to churches is associated with diagnosed HBP. Although there is no significant difference in proximity to mosques and diagnosed HBP, perhaps because mosques are randomly distributed not necessarily in response to population distribution. Thus, the observed association between the distribution of mosques and HBP is widespread in the study.

There was no association between distance from bus stops and the number of persons with HBP. There was no difference in distance to bus terminals between persons with HBP and those without it. All respondents are within 100 meters distance from non-major roads, which may imply that the neighbourhoods are walkable. Neighbourhoods with high walkability may ameliorate the risk of hypertension and this could play a significant role in improving population health. ^18^ However, persons with HBP live at a greater distance away from major roads (dual carriageway) compared with those who do have HBP and the number of persons with HBP decreases with increasing distance from major roads. There is an association between road infrastructure and HBP. Exposure to ambient PM_2_.5 concentrations from bus terminals can increase BP within a period of a few days while long-term exposure might also promote the development of chronic hypertension.^19^ There is an association between the level of traffic noise and increased blood pressure among cab drivers. ^20^

The random and dispersed patterns of churches and mosques respectively alluded to the lack of regulations guiding the establishment and approval of locations used by faith-based organizations in the study area and this lacuna exposed the majority of the population to noise pollution from these places of worship. Although churches and mosques have different distributional patterns, a greater number of people diagnosed with HBP live in their proximity. The random distributional pattern of mosques is particularly interesting because it shows that mosques are everywhere. Bus stops are dispersed because they are located in proximity to the road network. Therefore, both intermittent and continuous exposure to noise from religious centres and road networks can exacerbate the incidence of HBP among residents and this may worsen with the increasing density of religious centres in most African countries.

This study showed a strong, inverse relationship between distance to pharmaceutical shops, churches, mosques, road networks and the number of persons with HBP. Apart from noise from roadways, higher near-roadway particulate matter (PM_2.5_) and black carbon (BC) exposures have been associated with elevated BP.^21^ Although, primary care clinics are frequently placed in neighbourhoods to improve access.^22^ It is possible to infer that these facilities were not located in consonance with population distribution but rather based on available space. This further amplified the inadequate health infrastructural planning in most developing countries. The absence of a relationship could also be due to the combination of private and public healthcare facilities in the analysis. A similar study noted that the proximity of the home to hospitals did not correlate with primary immunization completion or BP in either a hospital-based or a community clinic.^22^

Noise from haphazardly distributed faith-based centres in the unplanned metropolitan area of Ibadan could predispose residents to HBP. noise levels are seldom studied at non-workplace and non-abode sites that are visited regularly, e.g., places of worship.^8^ Therefore, ambient noise levels emanating from religious activities in residential neighbourhoods are an emerging environmental problem that has attracted little attention from enforcement agencies and policymakers and components of such religious noise include, but are not limited to, mosque calls, ringing of church bells, megaphones, clapping of hands, loud prayers, chanting, singing, mobile preaching, open-air crusade, night vigils and drumming.^23^ Noise measurements outside some mosques in Saudi Arabia were higher than 85 dB which is the sound level at which noise-induced hearing loss (NIHL) has been shown to occur in occupational settings.^8^ Noise from religious centres has been recognized as a major source of sleep disturbances and may become unbearable with the increasing density of religious centres (churches and mosques) in the coming years.^24^ Apart from sleep disorders, the literature has established the link between noise exposure and HBP.^25–27^

The importance of the relationship between socioeconomic and housing variables to HBP was further established in this study. Comparatively, the likelihood of having HBP was higher among the unemployed, married, and divorced persons, while the likelihood of HBP increased with education, age group and income. Current attempts at addressing the persistently high number of persons with HBP have emphasised the need to improve the lifestyle of people with limited attempts at addressing their housing and environmental conditions. ^28^ The findings that single individuals fare better in terms of their BP compared to their married counterparts complement and build upon earlier research and corroborate other research findings in this regard. Research has linked marital conflict to heart rate and HBP in laboratory studies. ^29^ Nevertheless, there are pockets of studies where never married persons had a higher risk of HBP when compared to married persons, even after adjustment for different demographic, socioeconomic and lifestyle variables.^30^ Research also suggests that occupational characteristics contribute to adverse health outcomes such as CVD. ^31^ Persons of high occupational status are known to have higher BP, especially men.^32^ High psychological demand at the workplace, may contribute to disease risk, although, having control over one’s schedule and one’s work may mitigate the pathogenic effects.^33^ In contrast, ^34^ found a link between unemployment and worsening health conditions. Unemployment, increasing age, and low income have also been identified as cogent risk factors for HBP in this population and this has been reported in other studies. ^35^

## Figures and Tables

**Table 1:** Distributional Pattern of HBP, Health Care Facilities and Noise Generating Centres

Variables	Number	Observed Mean Distance	Expected mean Distance	Nearest Neighbor Ratio (Rn)	Z-Score	P-Value	Pattern
HBP Persons	1510	9.671413	25.094677	0.437726	41.799148	0.000000	Clustered
Health Care Facilities	17	270.383403	208.234018	1.298459	2.354185	0.018563	Dispersed
Pharmaceutical Shops	5	90.565038	383.964557	0.235868	3.268768	0.001080	Clustered
Churches	5	574.679866	383.964557	1.496700	2.124762	0.033606	Dispersed
Mosque	12	244.424270	247.848056	0.986186	0.091547	0.927058	Random
Bus Stops	3	718.580946	383.964557	1.871477	3.727966	0.000193	Dispersed

**Table 2:** Variations in Blood Pressure by Socioeconomic and Behavioural Characteristics

Variables	Options/Levels	Diagnosed HBP				
		No	Yes	Total	Chi-Square	Sig
Gender	Male	3117 (23.0%)	365 (2.7%)	3473 (25.7%)	2.085	0.079
	Female	8912 (65.9%)	1148 (8.5%)	10060 (74.3%)		
Occupation	Professional	623 (4.6%)	71 (0.5%)	694 (5.1%)	107.841	0.000
	Artisans	4491 (33.2%)	385 (2.8%)	4876 (36.0%)		
	Unemployed	629 (4.6%)	138 (1.0%)	767 (5.7%)		
	Others	6275 (46.4%)	919 (6.8%)	7194 (53.2%)		
Marital Status	Single	1868 (13.8%)	81 (0.6%)	1943 (14.4%)	308.067	0.000
	Married	8310 (61.4%)	962 (7.1%)	9228 (68.5%)		
	Divo rced/Separated	560 (4.1%)	116 (0.9%)	676 (5.0%)		
	Widowed	1280 (9.5%)	353 (2.6%)	1625 (12.1%)		
Education	No Formal Education	1157 (8.6%)	256 (1.9%)	1413 (10.4%)	164.773	0.000
	Primary	2225 (16.4%)	388 (2.9%)	2613 (19.3%)		
	Junior Secondary	659 (4.9%)	79 (0.6%)	736 (5.5%)		
	Senior Secondary	6771 (50.0%)	631 (4.7%)	7402 (54.7%)		
	Tertiary	1206 (8.9%)	158 (1.2%)	1364 (10.1%)		
	Others	0 (0.0%)	1 (0.0%)	1 (0.0%)		
Diabetes	No Diabetes	11930 (88.6%)	1464 (10.9%)	13394 (99.5%)	181.342	0.000
	Have Diabetes	28 (0.2%)	44 (0.3%)	72 (0.5%)		
Previous Stroke History	No Stroke History	11942 (88.7%)	1478 (11.0%)	13420 (99.7%)	135.441	0.000
	Have Stroke History	16 (0.1%)	30 (0.2%)	46 (0.3%)		
Heart Disease	No Heart Disease	11954 (88.8%)	1507 (11.2%)	13461 (100.0%)	0.39	0.532
	Have Heart Disease	4 (0.0%)	1 (0.0%)	5 (0.0%)		
High Cholesterol (dyslipidemia)	No Dyslipidemia	11947 (88.7%)	1496 (11.1%)	13443 (99.8%)	38.898	0.000
	Have Dyslipidemia	11 (0.1%)	12 (0.1%)	23 (0.2%)		
Obesity	No Obesity	11953 (88.8%)	1502 (11.2%)	13455 (99.9%)	20.801	0.000
	Have Obesity	5 (0.0%)	6 (0.0%)	11 (0.1%)		
Risk of Stroke	No Risk of Stroke	11950 (88.7%)	1493 (11.1%)	13443 (99.8%)	67.605	0.000
	Have a Risk of Stroke	8 (0.1%)	15 (0.1%)	23 (0.2%)		
Nothing	No Sickness	2645 (19.6%)	1476 (11.0%)	4121 (30.6%)	3618.961	0.000
	Yes Sickness	9313 (69.2%)	32 (0.2%)	9345 (69.4%)		
Tobacco	No Tobacco	11597 (86.1%)	1430 (10.6%)	13027 (96.7%)	19.692	0.000
	Yes Tobacco	361 (2.7%%)	78 (0.6%)	439 (3.3%)		
Kolanut	No Kolanut	11343 (84.2%)	1381 (10.3%)	12724 (94.5%)	255.042	0.000
	Yes Kolanut	615 (4.6%)	127 (0.9%)	742 (5.5%)		
Alcohol	No Alcohol	13255 (79.2%)	1337 (9.9%)	11998 (89.1%)	131.259	0.000
	Yes Alcohol	1297 (9.6%)	171 (1.3%)	1468 (10.9%)		
Have Stroke	No Stroke	11920 (88.5%)	1443 (10.7%)	13363 (99.2%)	281.253	0.000
	Yes Stroke	38 (0.3%)	65 (0.5%)	103 (0.8%)		
Mild Stroke	No Mild Stroke	11913 (88.5%)	1431 (10.6%)	13344 (99.1%)	335.299	0.000
	Yes Mild Stroke	45 (0.3%)	77 (0.6%)	122 (0.9%)		

**Table 3:** Sociodemographic and Housing Characteristics of Respondents and the Odds of Diagnosed Hypertension

Sociodemographic and Housing Variables	Categories	Diagnosed HBP: n (%)	OR (95% CI)	AOR (95% CI)
Gender	Male	365 (10.5)	1.00	
	Female	1148 (11.4)	1.08 (0.95, 1.23)
Occupation	Professional	71 (10.2)	1.00	1.00
	Artisans	385 (7.9)	0.78 (0.59, 1.02)	1.16 (0.86, 1.57)
	Currently Unemployed	138 (18.0)	**1.96 (1.43, 2.67)**	**1.58 (1.11, 2.24)**
	Others	919 (12.8)	**1.40 (1.08, 1.82)**	1.30 (0.97, 1.74)
Marital Status	Never Married	81 (4.2)	1.00	1.00
	Currently Married	962 (10.4)	**2.70 (2.13, 3.41)**	**1.45 (1.11, 1.89)**
	Previously married	470 (20.4)	**6.08 (4.75, 7.78)**	**1.75 (1.29, 2.38)**
Level of Education	No Formal Education	256 (18.1)	1.00	1.00
	Primary	388 (14.9)	**0.78 (0.66, 0.94)**	**1.35 (1.11, 1.64)**
	Secondary	710 (8.7)	**0.41 (0.35, 0.48)**	**1.31 (1.07, 1.62)**
	Tertiary	159 (11.7)	**0.53 (0.42, 0.66)**	**1.64 (1.25, 2.16)**
Age (years)	<25	69 (3.4)		1.00
	25–29	131 (6.3)	**1.88 (1.39, 2.53)**	**1.72 (1.26, 2.34)**
	30–34	119 (7.1)	**2.13 (1.57, 2.90)**	**1.83 (1.33, 2.52)**
	35–39	117 (7.7)	**2.42 (1.78, 3.29)**	**2.01 (1.44, 2.79)**
	40–44	161 (11.2)	**3.62 (2.70, 4.85)**	**3.00 (2.18, 4.12)**
	45–49	137 (12.1)	**4.04 (3.00, 5.46)**	**3.31 (2.38, 4.60)**
	50–59	293 (20.3)	**7.76 (5.89, 10.21)**	**6.27 (4.61, 8.54)**
	>=60	486 (22.3)	**8.66 (6.66, 11.27)**	**6.94 (5.04, 9.57)**
Income	0–50,000	1303 (11.0)	1.00	
	50,001–100,000	166 (11.9)	1.06 (0.89, 1.27)	
	>100,001	44 (13.8)	1.24 (0.89, 1.72)	
Type of Housing Unit	Compound	31 (11.9)	1.00	
	Storey Building	528 (12.0)	0.90 (0.61, 1.35)	1.04 (0.69, 1.58)
	Bungalow	1147 (13.0)	0.95 (0.61, 1.47)	0.90 (0.58, 1.42)
	Face to face	832 (10.5)	0.78 (0.52, 1.15)	0.80 (0.53, 1.21)
	Hut	5 (50.0)	**7.51 (1.99, 28.31)**	**876 (2.17, 35.37)**

## Summary

### What is known about the topic?

- Spatial results demonstrate specific health regions that may be in greater need of public health efforts toward promoting cardiovascular fitness that are tailored to particular regional and cross-sectional requirements.
- Distance from home to hospital often have significant effect on hypertension showing people living further from the facilities or town centers seemed to be less hypertensive. Also, greater geographic accessibility to primary care was associated with a decreased risk of hypertension while better secondary care accessibility was associated with an increased risk of hypertension and untreated hypertension.
- It has been found that both systolic and diastolic blood pressure were affected by religious commitment and religious activities
- In Africa, religious noise pollution is gradually becoming rampant and a nuisance as most churches and mosques produced high levels of religious noise, which far exceeded the permissible noise levels of EPA.

### What this study adds

- Persons diagnosed with high blood pressure appeared clustered in certain localities in cities compared to others localities and that the hotspots of high blood pressure are concentrated around churches, mosques, and major roads.
- While the average distance travelled to hospitals varied between those diagnosed and those not diagnosed with HBP, nonetheless, there was no significant relationship between the number of persons diagnosed with HBP and distance from healthcare facilities. The average distance to pharmaceutical shops for persons diagnosed with HBP was not significantly lower than those without diagnosed HBP, however, a significant negative relationship exists between distance from pharmaceutical shops and the number of persons with diagnosed HBP.
- The average distance to churches was significantly lower among persons with diagnosed HBP compared to those without diagnosed HBP and there was a significant inverse relationship between distance from churches and the number of persons with diagnosed HBP. However, there was no significant difference between the distance from mosques among those with diagnosed HBP and those without, nevertheless, a significant negative relationship was observed between distance from mosques and the number of persons with diagnosed HBP.
- The average distance from major roads among persons with diagnosed HBP was significantly higher than for those without diagnosed HBP and a significant inverse relationship was noticed between distance from major roads and the number of persons with diagnosed HBP.
- Persons previously diagnosed of stroke, diabetes, and high cholesterol, and those who consumed tobacco, Kola nut, and alcohol are more likely to be diagnosed with HBP.
- The determinants of diagnosed HBP include occupation, marital status, education, housing type, and age.
